# Editorial: Psychedelic-assisted psychotherapies: from clinical trials to credibility

**DOI:** 10.3389/fnins.2026.1816932

**Published:** 2026-03-23

**Authors:** Malkanthi Evans, Andrew Charrette

**Affiliations:** KGK Science Inc., London, ON, Canada

**Keywords:** addiction, DMT, emotional processing, neuroimaging, psilocybin, psychedelic medicine, psychotherapy, PTSD

This Research Topic examined the state of psychedelic therapies for mental health disorders, with a focus on strategies for treatment optimization, including algorithm-informed approaches.

Contributors were invited to discuss psychedelic-based therapies for mental health disorders. Authors were encouraged to submit original research, perspectives or opinions, and reviews of clinical studies that contribute to the evidence base and have potential clinical applicability.

## Background

Recent research has renewed interest in serotonergic psychedelics as potential interventions to alleviate the escalating burden of mental health disorders. In this context, psychedelic-assisted therapies have demonstrated fast-acting and durable effects, with an encouraging safety profile in controlled settings, with a low potential for abuse. Studies have paired psychedelic drug delivery with, at a minimum, psychological support to mitigate potential risks associated with acute disorientation and heightened suggestibility induced by these drugs. As psychological intervention is inextricably intertwined with the safe delivery of psychedelic therapies and because these drugs cause an altered state of consciousness that all but precludes effect masking in clinical trials, the robust characterization of their impact in the clinic is complicated, littered with concerns and continues to cause fierce debate among experts. Risks to psychological safety are inherent to the treatments, driving the need for refined clinical protocols to mitigate the risk of psychological adverse reactions, reduce variability in patient responses, and ameliorate the risks associated with the heightened suggestibility a patient experiences during and following the acute effects of the drugs.

While the limitations of existing treatment options have spurred investigations into the applicability of psychedelic therapies for chronic and treatment-resistant psychological disorders, there remain significant unresolved issues pertaining to how these treatments should be regulated and the implementation of an ethical framework for their widespread delivery in practice. Research investigating psychedelic research is challenged by methodological limitations such as proper masking of clinical trials, assessment of expectancy effects and therapeutic allegiance, which can lead to reproducibility issues across clinical trial sites. These issues have begun to be addressed through innovative clinical trial designs and the assessment of masking effectiveness and therapeutic allegiance indices; however, the majority of available data still reflects a paucity of these measures. Additional challenges to the integrity of the research have arisen from ethical missteps in trial conduct. Together, these challenges have impaired the field's advancement and must be addressed in the design of future studies to facilitate progress. While regulatory approval of psychedelic-assisted therapy remains a key milestone for the field, advances toward the systematic characterization of the effects and risks of these interventions have established a solid evidence base on which future research can inform the generalizability for regulatory authorization.

To this end, a significant clinical study has been undertaken to develop psychedelic compounds as therapeutic interventions in major depressive disorder (MDD), treatment-resistant depression (TRD), post-traumatic stress disorder (PTSD), generalized anxiety disorder (GAD) and substance-use disorders. Early-phase studies have provided signals of rapid symptom improvement in these indications, with supportive mechanistic insights from neuroimaging and established risk-mitigation practices. However, adequately powered, multi-site trials using harmonized protocols that delineate the effect of the psychotherapy component of treatments and provide long-term follow-up are still required. Drug development efforts have advanced treatments for PTSD, TRD, and MDD to phase 3 studies, which remain ongoing, yet these studies may remain limited in their capacity to sufficiently address these issues. These larger studies will provide greater sample sizes and longer-term follow-up, but do not explore the impact of the psychotherapy on the intervention. This progress is meaningful, but additional research will be required to fully explore this critical issue.

As highlighted in the FDA's review of Lykos Therapeutics' new drug application for MDMA, the influence, importance and complexity of psychotherapy's effect on the efficacy of psychedelic drugs remains unknown. Other drug developers, such as Compass Pathways and the USONA Institute, have sidestepped this Research Topic by including only “psychological support” instead of a psychotherapy component. Moreover, some believe that the subjective experience of psychedelics is not a prerequisite for the therapeutic effects of serotonergic agonists and is altogether unnecessary. This is a particularly contentious issue as many highlight the subjective experience as a critical element of efficacy.

Despite significant advances in our understanding of the neurophysiological effects of psychedelics from imaging and pharmacology studies, there remains a limited understanding of the complex mechanisms of action of these drugs. With the field still in its infancy, optimizing posology and treatment delivery remains at the forefront to ensure the generalizability of treatment effects. This collection of Research Topic provides the reader with a valuable glimpse into the state of the field, highlighting many of the significant issues that remain to be adequately addressed before psychedelic therapies can be considered a bona fide treatment for numerous mental health disorders.

A mini-review by Urban et al. identifies how psychedelics affect neural circuits involved in reward processing, stress regulation, and executive control, while also promoting forms of neuroplasticity that may reverse addiction-related neuroadaptations. They cite compelling evidence for the neurobiological mechanisms through which classical psychedelics may help treat substance use disorders. The literature gaps identified and the hypotheses established in the review will need to be addressed in future human studies before the relevance of the proposed mechanisms can be properly adjudicated.

Pharmaco-imaging by Soares et al. builds on this mechanistic perspective and investigates the acute effects of DMT on functional connectivity within networks involved in social cognition and emotional processing through a within-subject active-vs.-control design. They conclude that DMT modulates brain connectivity in socio-emotional and affective-value circuits in line with the hypothesis that psychedelics alter large-scale integration within the brain. The study is limited by a small sample size and the temporal constraints of imaging, which prevented the capture of onset dynamics. However, the study advances our understanding of the neural mechanisms that underlie the psychedelic experience and its potential therapeutic action.

Hooper et al. demonstrate that the aesthetic qualities of psychedelic experiences, such as perceptual richness, emotional resonance, and felt beauty, are strongly associated with emotional breakthroughs, psychological insight, and improvements in mental health. Subjective experience itself emerged as a critical determinant of clinical outcomes in psychedelic therapy. The authors introduce a novel construct: the aesthetic aspects of psychedelic experiences are not merely perceptual enhancements. They contend that aesthetic aspects may actively contribute to therapeutic outcomes. They recognize the limitations of the present study design and the potential for bias and conclude that further research is needed to validate their novel measure of aesthetic experience. Causal mechanisms can be further explored with longitudinal and experimental designs.

A broad, integrative review by Min et al. investigates the history, mechanism of action, production, and clinical applications of psilocybin. Their narrative synthesis presents evidence across multiple psychiatric disorders, including depression, PTSD, anorexia nervosa, and cluster headaches. The authors highlight psilocybin's capacity to promote lasting emotional openness, cognitive flexibility, and meaning-making. This article provides a valuable introduction to the use of psilocybin in mental health for the non-specialist, orienting the reader to the practical clinical considerations of the therapy and flagging unresolved issues in the field, such as long-term safety and protocol shortcomings.

One of the central questions facing the field is addressed by Zamaria et al., namely, what role should psychotherapy play in psychedelic-assisted treatment? They assert that psychedelic-assisted therapy is fundamentally a combined intervention in which pharmacological and psychotherapeutic components work synergistically. They state that therapeutic alliance, relational attunement, and integration practices significantly shape patient outcomes and safety. These authors contend that psychotherapy should be seen as essential to ensuring that psychedelic-induced insights and emotional experiences translate into lasting therapeutic change. While the opinion of the authors conveyed provides helpful guidance about the direction the field should move, the data available to tease out the contribution of psychotherapy in the intervention remains limited. The debate over the role of psychotherapy can only be addressed through rigorous experimental assessment. While not explored in detail in this article, further consensus on the protocol design elements and metrics required to provide conclusive evidence of the role of psychotherapy is a practical next step.

The examination of this Research Topic provides a narrative that undergirds the foundational theory of psychedelic-assisted therapy, which has converged over decades of research spanning the work of Humphrey Osmond, Stanislav Grof, and Roland Griffith, among others. In short, neurobiological plasticity catalyzed by psychedelics creates therapeutic potential but requires direction. Psychotherapy serves to shepherd these changes in neural signaling, thereby affecting shifts in mood and behavior to the benefit of the patient. The effectiveness and durability of the combination of the pharmacological intervention paired with psychotherapy is reliant upon the intrinsic meaningfulness associated with the non-ordinary state that psychedelics induce, which is shaped by the interpersonal context framed by the patient and therapist(s).

As psychedelic-therapy research has moved through the stages of commercialization, a departure from the traditional dogma of psychedelic therapy has emerged, which questions the necessity of both the altered state and the role of psychotherapy in the efficacy of psychedelic-assisted therapy. The implications of these questions are not trivial when considering real-world applications of these therapies, which, under the current model, are costly and inaccessible to many patients without financial support. Scientifically, questioning the established tenets of a novel treatment is healthy and should not be viewed with contempt.

These recent suggestions place the onus on future research to tease out the influence that psychotherapy and the subjective experience have on the effectiveness of psychedelic-assisted therapy while further characterizing the neurobiological and behavioral changes that occur. Likewise, the field still lacks decisive causal evidence that the putative plasticity mediators are in fact necessary or sufficient to provide the efficacy observed in mood disorders and addiction, and whether the results of healthy person trials translate to patients with these afflictions.

Collectively, the publications in this Research Topic underscore that progress toward clinical applicability and credibility will require methodological rigor and long-term follow-up to assess the persistence and generalizability of therapeutic effects over time, as well as the psychological safety of patients. As the field moves toward an era in which psychedelic-assisted therapy is increasingly implemented in clinics, the optimization of treatment protocols to improve efficacy and access will depend on research like that presented herein.

